# Brain‐Wide Measurement of Antibody Therapeutic Biodistribution and Target Engagement

**DOI:** 10.1002/alz70859_103765

**Published:** 2025-12-26

**Authors:** Chase Redd, Nathaniel Guanzon, Yessenia Gallegos, Camilla Stampe Jensen, Sandra B. Vergo, Damian G. Wheeler

**Affiliations:** ^1^ Translucence Biosystems, Inc, Irvine, CA USA; ^2^ H. Lundbeck A/S, Valby Denmark; ^3^ Activity Signaling, LLC, San Diego, CA USA

## Abstract

**Background:**

To look deep inside tissues, traditional histological methods cut specimens into thin slices. Providing access to the intricate anatomy of intact organs, tissue clearing offers neuroscientists unbiased and complete views of brain anatomy and function. One area where these methods have particular utility is in the development of CNS therapeutics where they can be used to examine the regional distribution of the therapeutics in the brain. We have developed a pipeline that provides unbiased and complete cellular resolution measurements of brain‐wide therapeutic biodistribution in pre‐clinical rodent brains.

**Methods:**

Using our iDISCO‐based tissue clearing method and a light sheet microscope, we can image micron‐scale resolution immunoreactivity across entire intact mouse brains. Here we explore the use of our technology to detect antibody therapeutics crossing the blood‐brain barrier (BBB) and engaging with targets.

**Results:**

In initial studies, we used a bispecific antibody engineered to bind to the Alzheimer’s Disease target, BACE1, as well as the transferrin receptor (TfR1), which helps shuttle the antibody across the brain endothelium and into the brain parenchyma. The IV‐dosed monospecific antibody could not be detected in the brain, but the bispecific Brainshuttle antibody was detected in the brain vasculature, presumably bound to TfR1, but also in the parenchyma, enriched in brain regions with high BACE1 expression, indicating that the bispecific antibody crosses the BBB and engages the target. To quantify the effective parenchymal levels of the bispecific antibody, we have extended our AI‐powered quantification pipeline to segment the staining in the vasculature and measure only the therapeutically relevant bispecific antibody signal in the brain parenchyma. Interestingly, the Brainshuttle mechanism is not always necessary. IV‐dosing of a Donanemab (Kisunla™) biosimilar was not detected in WT mice, but it accumulated in the brain of ARTE10 AD model mice, crossing the BBB and colocalizing with Amyloid plaques. Using our whole‐brain quantification tools, we segmented and quantified Donanemab biosimilar aggregates throughout the brain.

**Conclusion:**

These data provide a clear demonstration of the utility of tissue clearing methods for quantitative brain‐wide monitoring of Alzheimer’s Disease therapeutic antibody biodistribution.

